# Learn to be happy—an experimental study in clinical context with depressive patients in Germany

**DOI:** 10.3389/fpsyg.2024.1426597

**Published:** 2024-10-17

**Authors:** Elena Renée Sequeira-Nazaré, Bernhard Schmitz

**Affiliations:** Department of Psychology, Technical University Darmstadt, Darmstadt, Germany

**Keywords:** psychology, depression, positive, psychotherapy, well-being, happiness, satisfaction, patients

## Abstract

The increase in the number of people with mental disorders and the relapse rate of depressive patients give reason to constantly question and further develop psychotherapeutic interventions in order to stabilize therapy effects. Studies show that the art of living, described as the ability to lead a conscious and reflective life, can be learned and trained. The question arises as to what role the development of “art of living skills” have played in the treatment of depressed patients to date, and to what extent the targeted promotion of art of living within the framework of the therapy of depressed patients has an effect on the well-being and the level of depression that goes beyond this. The study is based on a mixed design. Study participants in the first experimental group (EG1, *n* = 53) each received one session of 50 min psychotherapy per week for 4 weeks. The second experimental group (EG2, *n* = 54) received psychotherapy equivalent to EG1 with additional life-skills reflection questions, which were to be answered every day and recorded in a diary. The control group (*n* = 53) received neither therapy nor reflection questions. The art of living, degree of depression, and satisfaction with life were assessed before and after the 4-week therapy and in the follow-up after 3 months, and the effects were compared. There was a significant decrease in the depression score over the 4 weeks in both therapy groups. EG2 showed a greater decrease in depression over the 4 weeks. However, the difference did not persist over the 3 months. Furthermore, in EG2 there was a significant increase in the Art of Living, Satisfaction with life, and Flourishing Scale over the 4 weeks, while in EG1 there was no change. However, the comparison in the follow-up after 3 months also shows that these effects could not stabilize. Overall, the results provide promising indications for integrating the art of living as a concept more strongly into the therapy of depressive patients. The question arises as to what long-term effects result from additional life skills stimulation and how the therapy effects gained can be stabilized over a longer period of time.

## 1 Introduction

In 2015, nearly one in four male and one in three female adults in Germany between the age of 18 and 79 suffered from a mental disorder (Gühne et al., 2015). Numerous studies prove the effectiveness as well as the long-term effect of psychotherapy with almost all medium to high effect sizes (Beutel et al., 2005; Ehrenthal, 2017; Franz et al., 2000; Huber et al., 2009). The relevance of psychotherapeutic support and research is increasing. Various effective factors can positively influence psychotherapy. These include, for example, a secure attachment style of the patient as well as pursuing a profession, whereby the profession itself does not seem to have a significant influence (Herrmann and Huber, 2013). There are also contradictory results regarding the duration of treatment (Borgart and Meermann, 1999) have found positive effects of treatment duration on treatment outcome, whereas other research, such as Fliege et al. (2002) and Franz et al. (2000) could not determine any influence of the treatment duration on the therapy success. One of the most important factors in psychotherapy is the patient-therapist relationship (Grawe, 1988). Herrmann and Huber (2013) investigated in a naturalistic study with 1,126 patients over 2 years the effect factors of inpatient psychotherapy with the help of a symptom check list. The results clearly show that occupation, patient motivation and younger age have a positive influence on successful therapy. Inpatient psychotherapy usually involves a combination of different therapeutic measures. Psychodynamically or behaviorally oriented individual psychotherapy, group therapy, couple and family discussions, medical visits and creative procedures such as art therapy, music therapy and body therapy procedures (in individual and group settings) are combined (von Wietersheim et al., 2020). In a research of von Wietersheim et al. (2020) the effects of inpatient therapy were investigated on the basis of 577 patients. This showed that there was a significant reduction in depression scores between admission and discharge. Depressiveness at the 3-month catamnesis after discharge initially increased again somewhat, but fell back to the discharge value at the 12-month catamnesis.

Koen Demyttenaere reports that, from the patients’ point of view, a rapid restoration of a normal experience of positive emotions, such as enjoyment of life, motivation and interest, is more important than alleviating depressed mood (Abdol, 2017). The desire grows to support well-being and satisfaction with life (Seligman et al., 2006). In contrast, improvements in negative emotions and depressed mood are among the most important goals of therapy for treating physicians (Demyttenaere et al., 2015). The importance of well-being in the context of mental disorders is supported by studies such as Wood et al. (2010) demonstrating that increased well-being reduces the risk of developing mental illness and has a symptom-relieving effect. Since the loss of positive emotions cannot be measured with common depression scales, it is often neglected in treatment decisions. Therefore, it is important to pay attention to patient-centered therapy (Abdol, 2017). In a study of Kennedy et al. (2016), the effect of the drug agomelatine in depressed patients over a period of 6 months was shown. Compared to other antidepressants, this drug also influences anhedonia and thus the gain of joy of life. The results after treatment with agomelatine showed significant improvements for the domains work and social and family life (Stiefelhagen, 2017). Another study showed similar results, with depressed patients with severe anhedonia improving from an initial sum score of 93.1–19.8 after 12 weeks of therapy (Vinckier et al., 2017). According to Schmitz (2016), art of living describes a good way of dealing with the self as well as a conscious, self-determined and reflective way of living. In psychotherapy it is worthwhile to deal with the art of living, because well-being is more than the absence of illness and therefore the treatment of depressive symptoms alone is not enough to be happy (Abdol, 2017). The components of the art of living include the self, the body, the soul, the environment and the mind. The latter includes the ability to reflect or self-reflect, which involves thinking about oneself, understanding one’s actions, examining them, evaluating them, and changing them if necessary (Christmann, 2003). From conclusions of self-reflection, one can set goals for the future that fit one’s own abilities and competencies and through which one can change and develop. Various studies indicate that the ability to self-reflect is related to increased well-being, and it is always crucial to generate alternative actions for the future (Lyubomirsky et al., 2011). Current studies indicate that existing treatment approaches have not yet succeeded in slowing down the rapid development of mentally ill people (Glied and Frank, 2009). Initial research approaches are yielding promising results for the application of positive interventions in clinical settings. For example, there is the application of so-called positive psychology interventions (PPI) (Sin and Lyubomirsky, 2009). These are exercises on topics such as gratitude, optimism, or forgiveness. Moderators of the effect of depressive symptom relief with PPI are the current depressive status, the severity of the depressive episode, the age of the patient, and the format and duration of the interventions (Bolier et al., 2013). The assumptions of Positive Psychotherapy (PPT) are built on the fact that people tend to remember, notice, and expect negative aspects more than positive aspects, which gives rise to negative emotions. This tendency is especially strong in depressed people. Because of this, exercises should be used to try to focus attention, memory and expectations on positive aspects (Seligman et al., 2006).

## 2 Methods

### 2.1 Study design and participants

The study is based on a two-factorial experimental mixed design, consisting of comparisons between three groups over the therapy period of 4 weeks and after 3 months, respectively. The experimental intervention, as an independent variable, was varied between groups. Study participants in the first experimental group (EG1) each received one session of 50 min per week of psychotherapy for 4 weeks. The second experimental group (EG2) received psychotherapy equivalent to EG1 and in addition life-skills reflection questions, which were to be answered every day and recorded in a diary. The control group (CG) received neither therapy nor reflection questions. Exclusion criteria for all groups were being a minor and having a BDIV score below the cut-off score of 35. For the control group, all participants were required to not currently be in psychotherapeutic treatment. Group assignment was randomized. A total of 20 psychotherapists participated in the study as investigators, each treating patients from EG1 or EG2. In terms of repeated measures, art-of-living, level of depression, satisfaction with life, and flourishing were assessed before and after the 4 weeks and in the follow-up after 3 months. The questionnaires used were the Art-of-Living Inventory (AOL), Flourishing Scale (FS), Satisfaction with Life Scale (SWLS), and Beck Depression Inventory V (BDI-V). With an effect size of ηp^2^ = 0.06 (corresponds to an f of about.253) and a power of.8, a total of 156 subjects was needed to determine a significant interaction effect with a mixed ANOVA with three groups and two measurements (*α* = 0.05). The sample of the two experimental groups consisted of voluntary patients from an inpatient psychiatric clinic and from different psychiatric outpatient clinics, who suffered from at least mild depression. The control group consisted of voluntary participants who were recruited via social media, among others, and who also suffered from at least mild depression (BDI ≥ 35). The total sample consisted of 161 volunteer study participants, 58% female and 41% male. There was one gender-neutral person. In total, there were 53 individuals in EG1, 54 in EG2, and 53 in CG. The groups did not differ significantly in gender composition (EG1: 62% female; EG2: 67% female; CG: 47% female) or age (EG1: M = 44.17 years, SD = 20.05 years; EG2: M = 43.06 years, SD = 15.11 years; CG: M = 40.98 years, SD = 20.05 years). In EG1, 62% had one or more pretreatments, in EG2, 61% and in CG 11%. In EG1, 47% of the study participants took medication, in EG2 50% and in CG 0.04%. The youngest study participant was 19 years old, and the oldest was 85 years old.

### 2.2 Instruments

The BDI is one of the earliest self-report scales for recording depressive symptoms/screening depressive disorders. The Depression Questionnaire (BDIV) consists of a total of 20 items, which are measured using a five-point Likert scale (0 = never, 5 = almost always). Cronbach’s alpha is 0.95 (Schmitt et al., 2006).

The questionnaire on the art of living consists of a total of 11 subscales with 35 items. It is the short form of a validated long-format questionnaire consisting of 131 items. The subscales are a self-determined way of life, self-knowledge, enjoyment, caring for the body, positive attitude to life, reflection, meaning, serenity, optimization, coping, and social contacts. The items are measured using a six-point Likert scale (1 = strongly deviating, 6 = strongly agreeing). The Cronbach’s alpha for the full scale was 0.92 (Schmitz et al., 2022).

The Flourishing Scale is a brief eight-item measure of the respondent’s self-perceived success in important areas of life such as relationships, self-esteem, purpose, and optimism. The scale provides a single psychological well-being score and can be used to provide useful feedback for how to improve one’s life and may stimulate self-reflection. The items are measured on a seven-point Likert scale (7 = strongly agree, 1 = strongly disagree). The test has a Cronbach’s alpha of 0.87 (Diener et al., 2010).

The five-item satisfaction with life scale is used to measure satisfaction with life (Janke and Glöckner-Rist, 2012). According to Pavot et al. (1991), this is a multifactorial construct with affective and cognitive-evaluative components. The affective components are characterized by the presence of positive and the absence of negative emotions. The cognitive-evaluative components consist of global and domain-specific satisfaction in various areas of life. The items are measured on a seven-point Likert scale (7 = strongly agree, 1 = strongly disagree) (Janke and Glöckner-Rist, 2012). Cronbach’s alpha is 0.85 (van Beuningen, 2012).

### 2.3 Procedures

Cognitive-behavioral psychotherapy in an individual setting served as an intervention for EG1 and EG2. Contents of psychotherapy include psychoeducation, behavioral analysis, emotion management, and confrontation exercises (Härter et al., 2017). The EG2 was given additional reflection questions, which the experimenter had based on the book (Schmitz, 2016). These consisted of the following questions: Reflection on today: (1) What did I enjoy today (even if only in a very small way)? (2) What am I proud of today (even if only in a very small way)? (3) What am I grateful for today? (4) Assuming I could relive the day, would I do something differently? If so, what would it be? Reflection on tomorrow: (5) Assuming I have all the power in the world tomorrow to do what I set out to do, what would it be? (6) How would I feel about it? and (7) How would people close to me notice that I was doing better? Participants of EG2 had to answer these questions every evening for a period of 4 weeks and write them down in a diary or similar.

### 2.4 Hypotheses

*H1: The depression level of EG1 and EG2 decreases significantly in the before-after comparison over the 4 weeks, while the depression level in the control group remains stable.*

*H2: Art of living, sociopsychological well-being, and satisfaction with life increase significantly in EG1 and EG2 over the 4 weeks, while no changes are seen in the control group.*

*H3: Thereby, EG2 shows a significantly higher increase in life art, satisfaction with life and subjective psychological well-being over the 4 weeks compared to EG1 and CG, as well as a significantly lower depression level.*

*H4: At the 3-month follow-up, depression and well-being scores of EG2 remain more stable than those of EG1.*

### 2.5 Analysis

**Figure 1:**
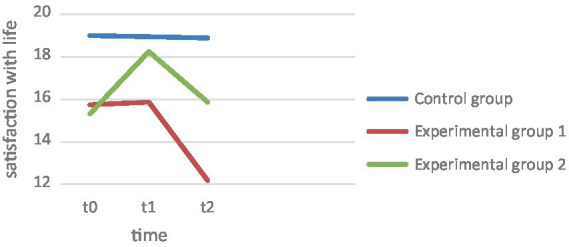
Satisfaction with life.

A one-factor ANOVA was first used to compare baseline scores. Across the three groups, the groups did not differ significantly in depression level *F* (2, 158) = 1.66, *p* = 0.194, while the group differences were significantly different in life skill variables *F* (2, 158) = 4.75, *p* = 0.010, Flourishing Scale *F* (2, 157) = 7.180, *p* = 0.001, and well-being *F* (2, 157), *p* = 0.003. In this regard, EG1 showed the lowest baseline scores at the descriptive level. To examine the extent to which variables changed within and between groups, multifactorial ANOVA were calculated across the three measurement time points, with group membership as the between-factor. Due to sphericity violation, the Greenhouse–Geisser correction is reported. Figure 1 shows the changes in the groups in the variables collected. Because of differences in baseline values, *post-hoc t*-tests for independent samples were used to compare the difference values/effects between groups to answer the hypotheses. The use of medication (0 = no, 1 = yes) and previous psychotherapeutic treatment (0 = no, 1 = yes) as independent variables had no significant influence on the difference effects of the dependent variable at any time.

### 2.6 Correlations

Table 1 shows the correlations between the variables at time t0. As expected, the depression score measured by the BDI shows a negative correlation with satisfaction with life and life skill. In contrast, the Flourishing scale shows almost no correlation with Depression. Furthermore, depression correlates positively with the number of pretreatments and medication use.

**Table 1:** Intercorrelation of the variables “depression, art of living, flourishing and satisfaction with life” collected over the three measurement times (*N* = 161).

Variable	*2*	*3*	*4*	*5*	*6*	*7*
Depression	−0.476**	0.579**	−0.529**	0.134	0.179*	0.202*
SWL		0.823**	0.708**	−0.103	−0.185*	−0.134
FS			0.797**	−0.160*	−0.164*	−0.114
AOL				−0.154	−0.080	−0.138
Age					0.206**	0.259**
Pretreatments *(0 = no, 1 = yes)*						0.631**
Medication *(0 = no, 1 = yes)*						

*p ≤ 0.05. **p ≤ 0.01.

## 3 Results

### 3.1 Hypothesis 1

The descriptive results and trajectories are shown in Figure 1. ANOVA across the three measurement time points with BDI score as the dependent variable proved significant in the main effect, *F*(1.49, 232.33) = 32.56, *p* ≤ 0.001, np^2^ = 0.17. As seen in Figure 1, both experimental groups record a drop. Pairwise comparison of mean differences within a group showed significant changes in EG1 and EG2 over 4 weeks (t0-t1), EG1: *F*(2, 155) = 12.84, *p* = 0.023, EG2: *F*(2, 155) = 51.27, *p* < 0.001, as well as between the second and third measurement (t1-t2), EG1: *F*(2, 155) = 12.84, *p* < 0.001, EG2: *F*(2, 155) = 51. 27, *p* < 0.001. Comparing the changes over the 3 months (t0-t2), these also proved to be significant, EG1: *F*(2, 155) = 12.84, *p* < 0.001, EG2: *F*(2, 155) = 51.27, *p* < 0.001. Hypothesis 1, which states that the depression level in both therapy groups decreases significantly over the 4 weeks, can thus be confirmed. In the control group, however, there were no significant changes over the 4 weeks, *F* (2, 155) = 0.087, *p* = 0.686. Comparing the changes in the control group over 3 months, there were also no relevant effects *F* (2, 155) = 0.087, *p* = 0.836.

### 3.2 Hypothesis 2

**Figure 2:**
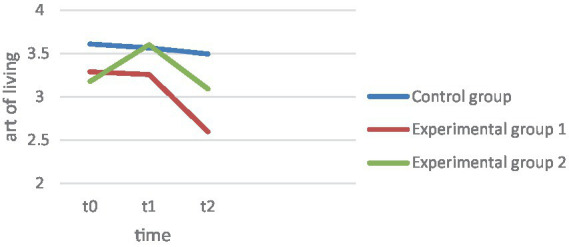
Art of living.

**Figure 3:**
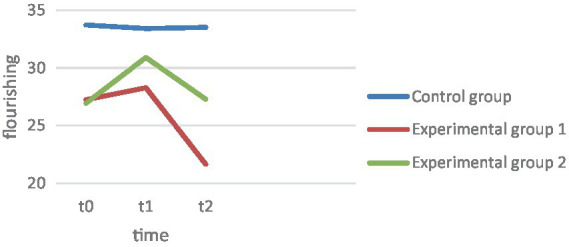
Flourishing.

Hypothesis 2 is concerned with the fact that life art, sociopsychological well-being, and satisfaction with life should increase significantly over the 4 weeks in EG1 and EG2. Art of Living: As seen in the graph of Figure 1, EG1 showed a slight decrease in life art over the 4 weeks, while EG2 showed an increase in life art (Figure 2). In the analysis of variance, the main effect of repeated measures was found to be significant, *F*(1.11, 176.09) = 16.40, *p* < 0.001, np^2^ = 0.09. The increase in art of living in EG2 was found to be significant over the 4 weeks, *F*(2, 157) = 49. 76, *p* < 0.001. In contrast, the changes in EG1 *F*(2, 157) = 10. 41, *p* = 0.465 as well as in CG *F*(2, 157) = 0.787, *p* = 0.299 proved not to be significant. Flourishing: ANOVA revealed the main effect to be significant, *F*(1.39, 212.03) = 10.00, *p* < 0.001, np^2^ = 0.06 (Figure 3). Over the 4 weeks, neither EG1 showed significant *F*(2, 152) = 11.05, *p* = 0.171 nor the control group *F*(2, 152) = 0.078, *p* = 0.925. In contrast, the increase in EG2 proved to be significant *F*(2, 152) = 14.11, *p* < 0.001. Satisfaction with life: On the other hand, the ANOVA with satisfaction with life as dependent variable proved to be significant, *F*(1.55, 242.22) = 9.05, *p* ≤ 0.001, np^2^ = 0.06. The changes in satisfaction with life showed in EG1 *F*(2, 155) = 9. 707, *p* = 0.828 as well as in the CG *F*(2, 155) = 0.007, *p* = 0.924 also showed to be non-significant, while they were significant in EG2, *F*(2, 155) = 14.91, *p* < 0.001. In this regard, hypothesis 2 can be confirmed for the changes in art of living, Flourishing Scale, and satisfaction with life for EG2. In contrast, the normal therapy group (EG1) did not show significant improvements in any of the variables.

### 3.3 Hypothesis 3

**Figure 4:**
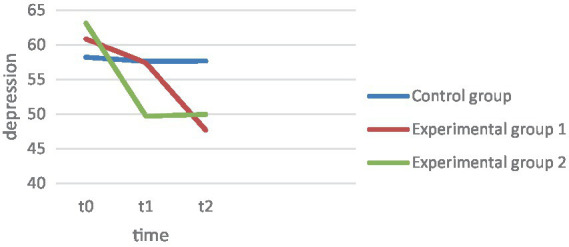
Depression.

To compare the change effects between groups, Hypothesis 3 predicts that EC 2 should have a significantly greater increase in life skills, sociopsychological well-being, and satisfaction with life over the 4 weeks, as well as a significantly greater decrease in depression levels. Depression: The interaction effect measurement*repeat group of the main analysis proved significant, *F* (2.98, 232.33) = 9.65, *p* ≤ 0.001, np^2^ = 0.11 (Figure 4). Tested by comparing difference scores, the decline in EG2 proved significantly greater than in EG1 *t*(104) = 6. 12, *p* < 0.001, *d* = 1.21. However, both experimental groups differed from the CG, EG1, *t*(157) = 4.02, *p* < 0.001, *d* = 0.63, EG2, *t*(104) = 5.50, *p* < 0.001, *d* = 1.1. Life skill: The interaction effect measurement repetition*group also proved significant with art of living as the dependent variable *F*(2.23, 176.09) = 5.7, *p* = 0.003, np^2^ = 0.07. Only in EG2 was there a descriptive increase in art of living, while art of living decreased in EG1. This difference also proved significant *t*(106) = −7.20, *p* < 0.001, *d* = 1.22. The art of living increase also differed significantly from the CG results, *t*(105) = −7.42, *p* < 0.001, *d* = 1.16, while art of living decreased equally in EG1 and CG, *t*(105) = −0.251, *p* = 0.401, *d* = −0.06. Flourishing: The interaction between group and measurement time points proved to be significant *F*(2.77, 212.03) = 5.28, *p* = 0.002, np^2^ = 0.07. Both experimental groups recorded an increase in Flourishing scale, but the increase in EG2 over the 4 weeks proved to be significantly higher, *t*(102) = −2. 81, *p* = 0.003, *d* = 0.55. While EG2 also differed from the CG over the 4 weeks *t*(101) = 4.20, *p* < 0.001, *d* = 0.72, there were no significant differences between the CG and EG1, *t*(105) = − 1.217, *p* = 0.113, *d* = 0.14. Satisfaction with life: For satisfaction with life, there was also an interaction between group membership and repeated measures, *F*(3.12, 242.22) = 5.34, *p* < 0.001, np^2^ = 0.06. Figure 1 graphically shows a strong increase in EG2 in satisfaction with life, which also proved to be statistically greater than in EG1, *t*(106) = −3. 17, *p* < 0.001, *d* = 0.61, as well as significantly larger compared to the CG, *t*(104) = −3.45, *p* < 0.001, *d* = 0.67. In contrast, the changes in EG1 did not differ from those in the control group, *t*(104) = −0.253, *p* = 0.400, *d* = 0.049. Thus, the results support the assumptions of hypothesis 3 in all variables. EG2 exhibits a significantly greater decrease in depression over the 4 weeks. While EG1 does not differ significantly from CG in the Positive Psychology variables, the effects of EG2 differ significantly from the other two groups.

### 3.4 Hypothesis 4

Hypothesis 4 assumes that the depression and well-being scores of EG2 will be more stable than those of EG2 at follow-up after 3 months. The extent to which within-group effects change at 3 months follow-up was tested first, and then the change effects between groups were compared. Depression: The BDI showed a significant decrease within both experimental groups after the 4 weeks, EG1, *F*(2, 155) = 12.838, *p* < 0.001, EG2, *F*(2, 155) = 51.126, *p* = 0.049. However, the decrease in depression did not differ between the experimental groups between t1 and t2, *t*(104) = −1.12, *p* = 0.268, *d* = 0.22, nor did it differ across the comparison over 3 months t0 vs. t2, *t*(106) = 1.43, *p* = 0.156, *d* = 0.28. Both experimental groups showed overall significant results over the 3 months, compared to the control group, EG1, *t*(159) = 4.73, *p* < 0.001, *d* = 0.84, EG2, *t*(105) = 5.48, *p* < 0.001, *d* = 1.06. Satisfaction with life: In satisfaction with life, EG2 showed a significant decrease in effects at follow-up after 3 months, *F*(2, 155) = 14.91, *p* = 0.005, so that EG2 did not show a significant gain in satisfaction with life in the comparison between t0 and t2, *F*(2, 155) = 14.91, *p* = 0.585. EG1 showed a sharp decrease in satisfaction with life over the course, so that the patients had significantly lower satisfaction with life after the 3 months than at t0 *F*(2, 155) = 9.707, *p* ≤ 0.001. The comparison of the mean differences of t0 and t2 between the experimental groups proved to be significant *t*(105) = −2.53, *p* = 0.013, *d* = 0.36, so that EG2 could record a significant satisfaction with life gain compared to EG1. Changes in EG1 and EG2 over the 3 months were not different from changes in the control group, EG1, *t*(157) = 1.12, *p* = 0.265, *d* = 0.21, EG2, *t*(103) = −0.49, *p* = 0.622, *d* = 0.65. Art of Living: In the art of living, EG2 also showed a significant decrease in effects at follow-up. *F*(2, 157) = 49.76, *p* < 0.001, showing no significant differences in life skills when comparing t3 and t0, *F*(2, 157) = 49.76, *p* = 0.574. Descriptively, it even shows that EG2 have a lower life skill level after 3 months. In EG1, there is no increase in life skill at all, but there is a sharp drop in follow-up, which also proved significant, *F*(2, 157) = 10.412, *p* < 0.001. Here, the drop in life skill after the 4 weeks is not significantly different between the experimental groups, *t*(106) = −0.57, *p* = 0.570, *d* = 0.11, however, EG1 shows a significantly higher drop when compared to t0, *t*(106) = −2.4, *p* = 0.019, *d* = 0.45. Comparing the changes with the control group over the 3 months, none of the experimental groups show significant differences from the control group, EG1, *t*(159) = 1.43, *p* = 0.154, *d* = 0.28, EG2, *t*(105) = −0.16, *p* = 0.873, *d* = 0.03. Flourishing: In EG2, the gained effects decrease significantly again after 3 months, *F*(2, 152) = 14.11, *p* = 0.017, as well as in EG1 *F*(2, 152) = 11.05, *p* < 0.001. Comparing t0 and t3, there is a strong significant decrease in EG1 below the baseline level of t0, *F*(2, 152) = 11.05, *p* < 0.001. Overall, the changes over the 3 months, t0-t3, are not different in EG1 and EG2, *t*(104) = −1.25, *p* = 0.106, *d* = 0.25. In fact, both experimental groups show a greater decrease in the flourishing scale over the 3 months than the control group, EG1, *t*(105) = 1.301, *p* = 0.098, *d* = 0.233, EG2, *t*(103) = 0.304, *p* = 0.001, *d* = 0.59. Hypothesis 4 cannot be confirmed with respect to depression. Both groups show a further decline at follow-up and do not differ significantly in the magnitude of the decline. Despite the decrease in effects after 4 weeks, EG2 could show a significantly higher satisfaction with life increase than EG1. Also, in the art of living, EG1 shows a significantly greater decline over the 3 months, so hypothesis 4 can be confirmed in terms of art of living and in terms of satisfaction with life.

## 4 Discussion

The results first show that in both therapy groups the depression level was significantly reduced over the 4 weeks, while the control group remained relatively stable over all three measurement time points, as expected. Overall, the scores of all dependent variables (depression, art of living, flourishing as well as satisfaction with life) of EG2 were worse than those of EG1 before therapy, while at follow up EG2 is above EG1 in all variables. After 3 months, depressive scores continue to improve in both experimental groups, with EG1 showing a higher decline at the descriptive level. In comparison, the improvement of EG2 flattens more after therapy. One reason for the higher therapy effects in EG2 could be the art of living intervention. This seems to lead to a stronger improvement of the depressive values during the therapy. It is possible that the improvement flattens out after the end of therapy, since no more intervention takes place, or that an extension of the intervention duration, e.g., 3 months instead of 4 weeks, might be more effective. The question arises whether the progress curves of EG1 and EG2 converge during longer observation or how they change further. In a study of Leichsenring et al. (2008), depressive patients were examined before therapy, after the end of therapy and after 1 year with regard to their depressive scores. The results showed that the majority of the subjects showed the greatest effect increase after 1 year compared to the end of therapy. For future studies, it is important to compare the therapy effects over a longer period of time in order to classify the effects in the long-term course.

In the art of living, EG1 shows a slight decrease after 4 weeks and a sudden decrease after 3 months. The reason for this could be the focus of the treating psychotherapists and physicians on symptom reduction (decrease of depression) instead of the restoration of positive emotions/joy of life (Demyttenaere et al., 2015). In comparison, one sees a strong increase in art of living in EG2. Thus, dealing with questions that stimulate the art of living, embedded in a therapy, could actually increase the art of living. After 3 months, however, there is also an extreme drop in the art of living, which is even just below the initial value. The reason for this could be a short-term deterioration in well-being, which is often found in patients after the end of therapy (Brakemeier et al., 2018). Some patients in the study reported after 3 months that the therapy had stirred up a lot in the aftermath and had thus led to a short-term worsening of symptoms and subjective well-being. They also reported that the therapist withdrawal and being on their own again had led to temporary worsening. However, most reported an improvement after overcoming the negative phase and after the “sore points” had been processed. Here, too, it would be interesting to observe the further course of events over a longer period of time.

In the positive variables of art of living, flourishing and satisfaction with life, EG1 shows no relevant improvements after 4 weeks and a sharp drop in follow-up after 3 months. This could be attributed to the focus of symptom reduction in depressed patients (Abdol, 2017). In comparison, EG2 showed a significant improvement in the art of living and satisfaction with life and flourishing after 4 weeks. Some patients reported that the questions stimulating the art of living were very difficult to answer at the beginning and were initially only answered superficially, with effects only becoming apparent after prolonged use. Thus, it is possible that the art of living itself is a learning process that basically takes more time. It is possible that patients need a subsequent therapy intervention that focuses on the training of the art of living in order to stabilize the effects.

This study has limitations. On the one hand, recruiting the test subjects was very difficult, since many patients were so stressed that it was an additional burden for them to have to fill out more questionnaires. Furthermore, there was a very high dropout rate, so that many only filled out the first questionnaire and were therefore not qualified for the study. The study was carried out in a paper pencil design because there were many older patients who would be overwhelmed with online questionnaires. As a result, however, there was massive paperwork and the patients often had to be reminded to keep to the other measurement times. Because many different therapists took part in the study, it was possible to ensure objectivity with regard to the experimenter, but it was not possible to check exactly whether the respective therapists carried out the test procedure in a similar way or followed the regulations.

Overall, the present study provides a versatile impetus for further interventions and research to increasingly integrate the concept of art of living into psychotherapeutic work. Obviously, the art of living plays an important role in people’s well-being and can help depressive patients to increase their satisfaction with life during and after therapy. Furthermore, it would be interesting for future studies to extend the art of living interventions and change the questions or the art of the intervention like a video based intervention for example. Also it is possible to test the intervention on various disorders apart from depression or to apply them in different therapeutic settings, e.g., in the context of a group therapy of depressive patients.

## Data Availability

The raw data supporting the conclusions of this article will be made available by the authors, without undue reservation.

## References

[ref1] Abdol A. A. (2017). Patientenzentrierte therapie: mehr lebensfreude und alltagskompetenz. InFo Neurol. 19:65. doi: 10.1007/s15005-017-2343-6

[ref2] Beutel M.E. Hoeflich A Kurth R Brosig B Gieler U Leweke F. (2005). Stationäre Kurz-und Langzeitpsychotherapie—Indikationen, Ergebnisse, Prädiktoren. In: Z. Psychosom. Med. Psychother. 51, 145–162. doi: 10.13109/zptm.2005.51.2.145 15931599

[ref3] Bolier L. Haverman M. Westerhof G. J. Riper H. Smit F. Bohlmeijer E. (2013). Positive psychology interventions: a meta-analysis of randomized controlled studies. BMC Public Health 13:S.119. doi: 10.1186/1471-2458-13-119, PMID: 23390882 PMC3599475

[ref4] Borgart E. J. Meermann R. (1999). Bedingungsfaktoren unterschiedlicher Behandlungsdauer bei Angststörungen im Rahmen stationärer Verhaltenstherapie. Psychother. Psychosom. Med. Psychol. 49, 109–113, PMID: 10373766

[ref5] Brakemeier E. L. Herzog P. Radtke M. Schneibel R. Breger V. Becker M.. (2018). CBASP als stationäres Behandlungskonzept der therapieresistenten chronischen Depression: Eine Pilotstudie zum Zusammenhang von Nebenwirkungen und Therapieerfolg. Psychother. Psychosom. Med. Psychol. 68, 399–407. doi: 10.1055/a-0629-7802 30286506

[ref6] Christmann U. (2003). Reflexivität: Rationalität, Theorieintegration zur Programmatik einer sozialwissenschaftlichen Psychologie. 2, 49–105.

[ref7] Demyttenaere K. Donneau A.-F. Albert A. Ansseau M. Constant E. van Heeringen K. (2015). What is important in being cured from depression? Discordance between physicians and patients (1). J. Affect. Disord. 174, 390–396. doi: 10.1016/j.jad.2014.12.004, PMID: 25545606

[ref8] Diener E. Wirtz D. Tov W. Kim-Prieto C. Choi D.-w. Oishi S.. (2010). New well-being measures: short scales to assess flourishing and positive and negative feelings. Soc. Indic. Res. 97, 143–156. doi: 10.1007/s11205-009-9493-y

[ref9] Ehrenthal J. C. (2017). Psychodynamische Psychotherapie – Grundlagen, Wirksamkeit, Methoden, Techniken. PSYCH up2date 11, 267–286. doi: 10.1055/s-0043-103157

[ref10] Fliege H. Rose M. Bronner E. Klapp B. F. (2002). Prädiktoren des Behandlungsergebnisses stationärer psychosomatischer Therapie. Psychother. Psychosom. Med. Psychol. 52, 47–55. doi: 10.1055/s-2002-20184, PMID: 11850853

[ref11] Franz M. Janssen P. Lensche H. Schmidtke V. Tetzlaff M. Martin K.. (2000). Effekte stationärer psychoanalytisch orientierter Psychotherapie—eine Multizenterstudie. Z. Psychosom. Med. Psychother. 46, 242–258. doi: 10.13109/zptm.2000.46.3.242, PMID: 11793315

[ref12] Glied S. A. Frank R. G. (2009). Better but not best: recent trends in the well-being of the mentally ill. Health Aff. 28, 637–648. doi: 10.1377/hlthaff.28.3.637 19414869

[ref13] Grawe K. (1988). “Beziehungsgestaltung in der Psychotherapie” in Der Mensch in der Psychiatrie. eds. Pfäfflin F. Appelt H. Krausz M. Mohr M. (Berlin, Heidelberg: Springer Berlin Heidelberg), 243–258.

[ref14] Gühne U. Becker T. Salize H. J. Riedel-Heller S. G. (2015). Wie viele Menschen in Deutschland sind schwer psychisch krank? Psychiatr. Prax. 42, 415–423. doi: 10.1055/s-0035-1552715 26540523

[ref15] Härter M. Schorr S. Schneider F. (2017). S3-Leitlinie/Nationale VersorgungsLeitlinie Unipolare Depression. Berlin, Heidelberg: Springer Berlin Heidelberg.

[ref16] Herrmann A. S. Huber D. (2013). Was macht stationäre Psychotherapie erfolgreich? Der Einfluss von Patienten-und Behandlungsmerkmalen auf den Therapieerfolg in der stationären Psychotherapie. Z. Psychosom. Med. Psychother. 59, 273–289. doi: 10.13109/zptm.2013.59.3.273 24085479

[ref17] Huber D. Albrecht C. Hautum A. Henrich G. Klug G. (2009). Langzeit-Katamnese zur Effektivität einer stationären psychodynamischen Psychotherapie. Z. Psychosom. Med. Psychother. 55, 189–199. doi: 10.13109/zptm.2009.55.2.189, PMID: 19402022

[ref18] Janke S. Glöckner-Rist A. (2012). Deutsche version der satisfaction with Life Scale (SWLS).

[ref19] Kennedy S. H. Avedisova A. Belaïdi C. Picarel-Blanchot F. de Bodinat C. (2016). Sustained efficacy of agomelatine 10 mg, 25 mg, and 25-50 mg on depressive symptoms and functional outcomes in patients with major depressive disorder. A placebo-controlled study over 6 months. Eur. Neuropsychopharmacol. 26, 378–389. doi: 10.1016/j.euroneuro.2015.09.006 26708320

[ref20] Leichsenring F. Kreische R. Biskup J. Staats H. Rudolf G. Jakobsen T. (2008). Die Göttinger Psychotherapiestudie. Forum Psychoanal. 24, 193–204. doi: 10.1007/s00451-008-0338-0

[ref21] Lyubomirsky S. Dickerhoof R. Boehm J. K. Sheldon K. M. (2011). Becoming happier takes both a will and a proper way: an experimental longitudinal intervention to boost well-being. Emotion 11, 391–402. doi: 10.1037/a0022575, PMID: 21500907 PMC4380267

[ref22] Pavot W. Diener E. Colvin C. R. Sandvik E. (1991). Further validation of the satisfaction with life scale: evidence for the cross-method convergence of well-being measures. J. Pers. Assess. 57, 149–161. doi: 10.1207/s15327752jpa5701_17 1920028

[ref23] Schmitt M. Altstötter-Gleich C. Hinz A. Maes J. Brähler E. (2006). Normwerte für das Vereinfachte Beck-Depressions-Inventar (BDI-V) in der Allgemeinbevölkerung. Diagnostica 52, 51–59. doi: 10.1026/0012-1924.52.2.51

[ref24] Schmitz B. (2016). Art-of-Living. Cham: Springer International Publishing (63).

[ref25] Schmitz B. Schumacher B. Schwarz M. Feldmann F. (2022). Validation of a German and English version of the revised art-of-living inventory. Eur. J. Psychol. Assess. 38, 124–136. doi: 10.1027/1015-5759/a000650

[ref26] Seligman M. E. P. Rashid T. Parks A. C. (2006). Positive psychotherapy. Am. Psychol. 61, 774–788. doi: 10.1037/0003-066X.61.8.774 17115810

[ref27] Sin N. L. Lyubomirsky S. (2009). Enhancing well-being and alleviating depressive symptoms with positive psychology interventions: a practice-friendly meta-analysis. J. Clin. Psychol. 65, 467–487. doi: 10.1002/jclp.20593, PMID: 19301241

[ref28] Stiefelhagen P. (2017). Depression: Gewinn an Lebensfreude für den Patienten sehr wichtig. DNP 18, 65–66. doi: 10.1007/s15202-017-1866-5

[ref29] van Beuningen J. (2012). The Satisfaction With Life Scale Examining Construct Validity. Netherlands: The Hague/Heerlen.

[ref30] Vinckier F. Gourion D. Mouchabac S. (2017). Anhedonia predicts poor psychosocial functioning: results from a large cohort of patients treated for major depressive disorder by general practitioners. Eur. Psychiatry 44, 1–8. doi: 10.1016/j.eurpsy.2017.02.485, PMID: 28535406

[ref31] von Wietersheim J. Knoblauch J. D. Rottler E. Weiß H. Hartmann A. Rochlitz P.. (2020). Therapeutischer Aufwand in stationärer oder tagesklinischer Behandlung und Therapieerfolg bei Patienten mit depressiven Störungen. Psychother. Psychosom. Med. Psychol. 70, 283–291. doi: 10.1055/a-1038-4708, PMID: 31822030

[ref32] Wood A. M. Froh J. J. Geraghty A. W. A. (2010). Gratitude and well-being: a review and theoretical integration. Clin. Psychol. Rev. 30, 890–905. doi: 10.1016/j.cpr.2010.03.005, PMID: 20451313

